# COVID-19: An Emerging Threat to Antibiotic Stewardship in the Emergency Department

**DOI:** 10.5811/westjem.2020.7.48848

**Published:** 2020-08-07

**Authors:** Michael S. Pulia, Ian Wolf, Lucas T. Schulz, Aurora Pop-Vicas, Rebecca J. Schwei, Peter K. Lindenauer

**Affiliations:** * University of Wisconsin Madison, School of Medicine and Public Health, Department of Emergency Medicine, Madison, Wisconsin; † University of Wisconsin Madison, School of Medicine and Public Health, Madison, Wisconsin; ‡ University of Wisconsin Madison, School of Medicine and Public Health, Department of Pharmacy, Madison, Wisconsin; § University of Wisconsin Madison, School of Medicine and Public Health, Department of Medicine, Madison, Wisconsin; ¶ University of Massachusetts Medical School - Baystate, Department of Medicine, Springfield, Massachusetts

## Abstract

While current research efforts focus primarily on identifying patient level interventions that mitigate the direct impact of COVID-19, it is important to consider the collateral effects of COVID-19 on antimicrobial resistance. Early reports suggest high rates of antibiotic utilization in COVID-19 patients despite their lack of direct activity against viral pathogens. The ongoing pandemic is exacerbating known barriers to optimal antibiotic stewardship in the ED, representing an additional direct threat to patient safety and public health. There is an urgent need for research analyzing overall and COVID-19 specific antibiotic prescribing trends in the ED. Optimizing ED stewardship during COVID-19 will likely require a combination of traditional stewardship approaches (e.g. academic detailing, provider education, care pathways) and effective implementation of host response biomarkers and rapid COVID-19 diagnostics. Antibiotic stewardship interventions with demonstrated efficacy in mitigating the impact of COVID-19 on ED prescribing should be widely disseminated and inform the ongoing pandemic response.

## BACKGROUND

Severe acute respiratory syndrome coronavirus 2 (SARS-CoV-2) is a novel viral pathogen and its associated clinical syndrome (COVID-19) is the cause of an ongoing global pandemic involving hundreds of thousands of deaths. While current research efforts focus primarily on identifying therapeutic interventions, it is important to consider the collateral effects of COVID-19 on other public health crises. Specifically, early reports suggest high rates of antibiotic utilization in COVID-19 patients despite their lack of direct activity against viral pathogens.^1^,^2^ Unnecessary use of antibiotics is a primary driver of antimicrobial resistance, a global public health crisis,^3^,^4^ and a significant risk to patient safety due to the risk of serious adverse drug events (ie, allergic reactions) and *Clostridioides difficile* infection.

As the hospital entry point for most patients with potential COVID-19, the emergency department (ED) is a critical setting for stewardship efforts.^5^ The ED has unique, systems-level barriers to quality improvement interventions that require customized approaches to antibiotic stewardship.^6^ It is important to consider how the ongoing pandemic exacerbates existing challenges to antibiotic stewardship for acute respiratory conditions in the ED.

## ANTIBIOTIC USE IN PATIENTS WITH COVID-19

Most available reports on COVID-19 have focused on characterization of the disease and associated outcomes; so there is limited information available on antibiotic use patterns. Two recent systematic reviews identified that 72% of patients with COVID-19 receive antibiotic therapy despite only 7% having a bacterial co-infection.^2^,^7^ While these findings raise substantial concern about potential overuse of antibiotics in COVID-19, the underlying studies are lacking sufficient prescribing detail to fully characterize the dilemma. Specifically, they do not include critical details such as when/where the antibiotics were initiated, indication for initiation (eg, empiric therapy vs confirmed co-infection), and duration/spectrum of therapy. In the few studies that did report co-infections, they often fail to provide details on how the infection was diagnosed and type of infection (ie, viral, bacterial, or fungal). There is a need to obtain prescribing data from the ED due to its primacy in the initial evaluation of COVID-19 patients and its susceptibility to the stewardship challenges posed by the pandemic. Only by gathering this information can the magnitude and appropriateness of ED-based antibiotic prescribing related to COVID-19 be accurately evaluated.

## POTENTIAL ADVERSE EFFECTS OF COVID-19 ON EMERGENCY DEPARTMENT STEWARDSHIP

Due to unique, system-level factors, optimizing antibiotic prescribing in the ED is challenging. Emergency care providers have specifically reported that time pressures, clinical inertia, perceived patient expectations, and diagnostic uncertainty can lead to overuse of antibiotics.^8^,^9^ Individual providers’ perceptions of the risk-to-benefit ratio for antibiotics may also drive the substantial inter-provider prescribing rate variability observed in the literature.^10^,^11^ In EDs experiencing a surge in volumes related to COVID-19 (eg, New York), the increased caseload may exacerbate the pre-existing barriers to stewardship. Additionally, fear of COVID-19 and a baseline lack of understanding among some patients of how antibiotics work may accelerate actual or perceived expectations for antibiotic therapy.^12^

These challenges in optimizing antibiotic prescribing in the ED are further exacerbated by diagnostic limitations inherent to the first pandemic wave. Patients with COVID-19 infection can present with a wide spectrum of illness severity and non-specific clinical features (eg, cough, dyspnea) that overlap substantially with other common acute respiratory conditions such as asthma, congestive heart failure, and bronchitis.^13^ Given the lack of widespread access to accurate and rapid COVID-19 diagnostics, including the absence of point-of-care assays, it is incredibly difficult to differentiate COVID-19 from other acute respiratory conditions for which antibiotics are generally indicated (eg, community-acquired pneumonia and chronic obstructive pulmonary disease exacerbations). This is likely to drive further overuse of antibiotics, given that high rates of unnecessary antibiotic prescribing are already observed for respiratory conditions of less diagnostic uncertainty (eg, asthma, influenza, and bronchitis), where therapeutic guidelines are clearly established and do not support antibiotic use.^14^–^16^

The increased prevalence of early hypoxia and progression to respiratory failure reported for COVID-19 compared to other infectious respiratory conditions is another potential factor underlying high rates of antibiotic utilization.^17^ For instance, Sepsis CMS Core Measure 1 (SEP-1) requires rapid administration of broad-spectrum antibiotics for all ED patients with two systemic inflammatory response syndrome criteria and a lactate > 2.0 mmol/L. This definition of severe sepsis may create antibiotic prescribing pressure for patients with minor vital sign or metabolic perturbations.^18^ Additionally, early guidelines for critically ill patients with COVID-19 recommended consideration of antibiotics due to the possibility of bacterial co-infection, despite a lack of evidence demonstrating improved outcomes.^19^,^20^

Although not directly related to COVID-19 infections, the pandemic public health response effort appears to have significantly altered patterns of ED utilization. Several reports confirm dramatic decreases in ED volumes for emergent conditions, potentially related to “stay at home” orders or fear of COVID-19 exposure in the hospital.^21^,^22^ Delayed presentations can cause more severe presentations of acute respiratory (eg, asthma) and infectious conditions (eg, sepsis), which may in turn lead to increased rates of antibiotic utilization and expanded spectrum of empiric therapy. While these effects are speculative, due to a lack of detailed reporting on antibiotic treatment of COVID-19 patients in the ED, they share similarities with known challenges to ED antibiotic prescribing and can be mitigated by established stewardship interventions.

## EMERGENCY DEPARTMENT ANTIBIOTIC STEWARDSHIP STRATEGES FOR COVID-19

There are several relevant stewardship interventions with established effectiveness in curbing inappropriate antibiotic usage for acute respiratory conditions in the ED. Academic detailing, care pathways/guidelines, and pharmacist review can improve empiric antibiotic selection for community-acquired pneumonia.^23^–^27^ The use of rapid, viral pathogen-detection assays has been proposed as a means to reduce antibiotic initiation and facilitate earlier discontinuation among ED patients with respiratory tract infections (eg, influenza).^28^–^31^ The ID NOW point-of-care testing platform by Abbott (Chicago, IL) that is used for rapid influenza, strep A, and respiratory syncytial virus now has a test for COVID-19 that can deliver a test result in 13 minutes with over 90% agreement with molecular polymerase chain reaction (PCR) assays for SARS-Cov-2.^32^,^33^ Given the role of diagnostic uncertainty in antibiotic prescribing, these assays will be an increasingly important tool for optimizing stewardship during the ongoing pandemic.

Although the availability, diagnostic performance, and turnaround time of diagnostic tests for SARS-CoV-2 is likely to continue improving over time, we are over five months into this pandemic and rapid testing is still not widely available in the ED. There is an immediate need for research elucidating the role of host response biomarkers in helping clinicians identify bacterial infections in patients with acute respiratory illnesses. Procalcitonin (PCT) is a Food and Drug Administration-approved biomarker that differentiates viral from bacterial infections and can safely guide antibiotic decision-making for stable patients with acute respiratory infections.^34^ However, rapid PCT is not widely used in the US and a recent trial demonstrated significant antibiotic use in low-risk pneumonia patients despite a negative PCT, suggesting the need for additional clinician education and decision support.^35^.^36^

Early reports indicate PCT remains negative in COVID-19 infection and may be useful in easing concerns of bacterial co-infection, although further research is needed to confirm these findings.^1^,^37^,^38^ Finally, although not yet available in the US, a point-of-care host response assay for respiratory tract infections that incorporates both a bacterial (C-reactive protein) and viral biomarker (myxovirus resistance protein A) has a reported 99% negative predictive value for bacterial infections.^39^ The finger-stick sample collection method and 10-minute turnaround time offer a promising alternative to PCR assays that require higher level personal protective equipment during nasal swab collection and are generally associated with turnaround times of several hours. This assay has been proposed as a potential triage tool in a tiered COVID-19 diagnostic strategy, but further validation of its performance in confirmed cases will be required before introduction into clinical practice.^40^

## CONCLUSION

The ongoing COVID-19 pandemic is exacerbating known challenges to optimal antibiotic stewardship in the ED, representing an additional direct threat to patient safety and public health via antibiotic overprescribing and promotion of bacterial resistance. There is an immediate need for research characterizing ED antibiotic prescribing patterns and the performance of host response biomarkers among patients with confirmed or suspected COVID-19. Antibiotic stewardship approaches shown to effectively mitigate the impact of COVID-19 in the ED should be widely disseminated and inform future pandemic responses.
